# Hawthorn Leaf Flavonoids Protect against Diabetes-Induced Cardiomyopathy in Rats via PKC-*α* Signaling Pathway

**DOI:** 10.1155/2017/2071952

**Published:** 2017-10-04

**Authors:** Qing Min, Yuting Bai, Yuchen Zhang, Wei Yu, Minli Zhang, Dongyang Liu, Tingting Diao, Wei Lv

**Affiliations:** Department of Pharmacology, School of Pharmacy, Hubei University of Science and Technology, Xianning 437100, China

## Abstract

**Objectives:**

DCM has become one of the main reasons of death in diabetic patients. In this study, we aimed to explore the hawthorn leaf flavonoids (HLF) protective effect against diabetes-induced cardiac injury and the underlying mechanisms in experimental rats.

**Methods:**

Experimental diabetic model was induced by intraperitoneal injection of streptozotocin (STZ, 40 mg/kg) in rats after feeding with high-fat diet for 8 weeks. The diabetic rats received a 16-week treatment of different doses of HLF (50, 100, and 200). The morphological changes of myocardial cells were observed by light microscope; the concentration of antioxidant indicator and TNF-*α* and the expression of PKC-*α* mRNA, PKC-*α*, and NF-*κ*B proteins were assessed as well.

**Results:**

STZ-induced diabetes mellitus prompted blood glucose, cardiac injury, oxidative stress, and inflammation, accompanied with suppressed body weight. On the contrary, HLF administration improved body weight and blood glucose and attenuated myocardial structural abnormalities in diabetic rats. In addition, HLF decreased MDA level and enhanced SOD activities, inhibited TNF-*α* expression, and downregulated PKC-*α* mRNA, PKC-*α*, and NF-*κ*B which were induced by diabetes.

**Conclusions:**

HLF has a protective effect against diabetic cardiomyopathy in rats. The mechanism may be involved in reducing oxidative stress and inflammation via inactivation of the PKC-*α* signaling pathway.

## 1. Introduction

The incidence of diabetes increased year by year and has become a major health threat to human. The International Diabetes Federation (IDF) predicts that the number of diabetic patients will reach 529 million by 2035 [1]. Researchers discovered that diabetes has a close relationship with heart failure and epidemiological studies revealed that people with diabetes are more likely to have heart failure [2]. Rubler put forward diabetic cardiomyopathy (DCM) initially in 1972 by autopsying the corpses of diabetic patients with heart failure [3]. DCM suggests a direct cellular injury to myocardium, which is independent of hypertension, coronary heart disease, and other cardiovascular diseases. Left ventricular hypertrophy, cardiac function drops, ventricular electrophysiological disorders, and heart failure are the major performance of DCM [4]. To date, DCM has become one of the main reasons of death in diabetic patients. However, there are still few effective strategies to prevent the development of DCM.

Flavonoids compounds, extracted from natural plant, such as nobiletin [5], breviscapine [6], and resveratrol [7], exert cardioprotective function through inhibiting the expression of PKC. In recent years, hawthorn leaf flavonoids (HLF), a natural flavonoid, received extensive attention domestically and overseas. Studies have reported that HLF possesses a broad spectrum of biological properties, such as anti-inflammatory [8] and antioxidant [9] and ameliorates hepatic steatosis [10] and anticancer [11] effects. Moreover, quercetin, a chemical component of HLF, protected vascular endothelial cells by inhibiting the expression of PKC [12, 13]. It is well-known that diabetes induced intracellular reactive oxygen species (ROS) accumulation and subsequently increased inflammation and apoptotic cardiac cell death. Therefore, we hypothesized that HLF could play a protective role against diabetes-induced cardiomyopathy.

In the present study, experimental diabetic animal model was induced by low dose of streptozotocin (STZ) combined with high-fat diet on rats. We found support for the hypothesis that HLF attenuates myocardial injury induced by diabetes and explored whether the effects of HLF are mediated through the PKC pathway.

## 2. Materials and Methods

### 2.1. Experimental Animals

Eighty male Sprague Dawley rats (90 ± 10 g) were purchased from Laboratory Animal Center of Wuhan University. All experimental protocols and procedures were conducted in accordance with the Guide for the Care and Use of Laboratory Animals published by the National Institutes of Health (NIH publication 85-23, revised 1996) and approved by the Committee of Experimental Animals of Hubei University of Science and Technology.

### 2.2. Induction of Diabetic Model and Treatment with HLF

The rats were randomly divided into control and diabetic model groups; control group rats (*n* = 10) were fed with normal diets while diabetic model group (*n* = 70) were fed with 8-week high-fat diet; after that, the diabetic model rats were intraperitoneally injected with STZ (dissolved in 0.1 mol/L citrate buffer, pH 4.5) at the dose of 40 mg/kg for just one time, and the control rats with an equivalent volume of citrate buffer. Blood glucose levels were measured using glucometer by tail vein puncture blood sampling after 72 h injection. Blood glucose levels > 16.7 mmol/L were used in this study. A model preparation of a total of 52 rats was successful, and then these rats were randomly divided into 4 subgroups: diabetic cardiomyopathy group (DCM, *n* = 16) and HLF groups (LHLF: low dose, 50 mg/kg, *n* = 12; MHLF: medium dose, 100 mg/kg, *n* = 12; HHLF: high dose, 200 mg/kg, *n* = 12). HLF was orally administrated to HLF groups once a day for 16 weeks. The control and DCM groups were treated with equal volume of saline.

### 2.3. Blood Glucose and Weight Determination

Blood glucose levels were measured by tail vein puncture blood sampling every 2 weeks; all rats fasted 12 hours before being measured. All animals were provided with food and water ad libitum and without insulin and other hypoglycemic agent during experiment.

### 2.4. Specimen Collection

At the end of experiment, hearts were excised and heart weight was assessed after anesthesia. The part of heart tissue was fixed with 4% paraformaldehyde for H&E staining. The other remaining heart tissues were stored at −80°C for biochemical assay, RT-PCR, or western blot assay.

### 2.5. Biochemical Assay

The samples of heart tissue were weighed and homogenized (1 : 9, w/v) in normal saline; then homogenate was centrifuged and supernatant was collected. The superoxide dismutase (SOD), malondialdehyde (MDA), and tumor necrosis factor-*α* (TNF-*α*) were measured using the associated detection kits according to the manufacturer's instructions in different reagent kits. SOD and MDA detection kits were purchased from Nanking Jiancheng Bioengineering Research Institute, China. TNF-*α* Elisa Detection Kits were purchased from Shanghai Biorui Biotech Company, China.

### 2.6. Histological Analysis

The samples of heart tissue were fixed with 4% paraformaldehyde for 24 h, then embedded in paraffin, and sectioned into 4 *μ*m thick samples for histological analysis. The sections were stained with Hematoxylin-Eosin (H&E) and photographed under light microscopy at ×400 magnification.

### 2.7. RT-PCR Analysis

Total RNA was extracted from heart tissue by using TRIzol reagent (Takara Bio, USA) and the RNA concentrations were determined by UV spectrophotometry. Oligo(dT) first-strand cDNA was synthesized from 4 *μ*g total RNA using reverse transcription kit (Fermentas, Hanover, MD, USA). GAPDH was used as an endogenous reference gene because of its uniform expression among all samples. Primer sequences used in real-time PCR are listed as follows: PKC-*α* (310 bp): 5′-CGTAGGAGTGTCCGTGGA-3′ (forward) and 5′-TCGGAAAACCATGTATCG-3′ (reverse), GAPDH (358 bp): 5′-CGGGAAGCTTGTCATCATCAATGG-3′ (forward) and 5′-GGCAGTTGATGGCATGGACTG-3′ (reverse). The amplification condition of PCR was as follows (in a total volume of 50 *μ*L): 1 cycle of 50°C for 2 min, 2 cycles of 94°C for 4 min, followed by 40 cycles of 94°C for 30 s, 54°C for 30 s, and 72°C for 25 s, then 1 cycle of 95°C for 15 s, and 1 cycle of 60°C for 1 min.

### 2.8. Western Blot Analysis

The membrane protein, cytoplasm protein, and nucleus protein were, respectively, extracted from heart samples by using RIPA lysis buffer and then the protein concentration was measured by Bicinchoninic Acid (BCA) protein assay (Beyotime Biotechnology, China). Equal proteins were separated by SDS-PAGE gels and then transferred to PVDF membrane, blocking the membrane with 5% nonfat milk, and the primary antibodies of PKC-*α*, NF-*κ*B p65, and H3 (CST, CA, USA) were used for western blot. The membrane was treated with appropriate secondary antibody for 1 h at 37°C. Finally, the protein band information was read using chemiluminescence system (Pierce Biosciences, USA) and then quantified the immunoblots using quality one image analysis software.

### 2.9. Statistics Analysis

Data were analyzed by using GraphPad Prism 5.0 and expressed as mean ± SD, all statistical analyses were subjected to one-way ANOVA, and differences were considered significant if *P* < 0.05.

## 3. Results

### 3.1. The General Condition of Diabetic Rats

After 16-week administration, eight rats died due to diabetic ketoacidosis, infection, or cardiomyopathy in DCM group, three died in low and medium dose HLT treatment group, and two died in high dose HLT treatment group. Furthermore, control group rat's fur was white and glossy. DCM group rat's fur was yellow and dark; about half of rats have diabetic complications such as cataract. Interestingly, after the treatment of HLF, these changes were reversed. For the low dose group, all symptoms were improved except mild cataract of individual rats, but the cataract was rarely seen in high dose group.

### 3.2. The Effect of HLF on Metabolism of Diabetic Rats

DCM group rats exhibited typical symptoms of diabetes including hyperglycemia, polydipsia, and polyuria. As shown in Table 1, the body weight and heart weight of DCM group rats were all significantly reduced compared with the control group (*P* < 0.05 versus control group), while the blood glucose levels and the heart-to-body weight ratio (HW/BW) were significantly increased in DCM group (*P* < 0.05 versus control group). After treatment by HLF for 16 weeks, compared with the DCM group, the body weight and heart weight of high dose group were significantly increased, and the blood glucose levels were decreased in all of HLF treatment groups and showed a dose dependence manner. Furthermore, the ratios of HW/BW in HLF medium dose group and high dose group were markedly reduced (*P* < 0.05 versus DCM group).

### 3.3. The Effect of HLF on MDA Content and SOD Activity in Myocardial Tissue of Diabetic Rats

As shown in Table 2, compared with the control group, the MDA content in myocardial tissue was significantly increased in DCM group (*P* < 0.05 versus control group), while the SOD activity in DCM group was decreased but exhibited little difference between control and diabetic group. Compared with the DCM group, HLF treatment significantly decreased the level of MDA and increased the activity of SOD of heart especially in HLF medium and high dose treatment group (*P* < 0.05 versus DCM group).

### 3.4. The Effect of HLF on TNF-*α* in Myocardial Tissue of Diabetic Rats

As shown in Table 3, compared with the control group, the level of TNF-*α* in myocardial tissue of DCM group was significantly increased (*P* < 0.05 versus control group). However, the level of TNF-*α* was markedly inhibited after treatment with HLF even in a low dose of HLF (*P* < 0.05 versus DCM group).

### 3.5. The Effect of HLF on Myocardial Cell Morphology of Diabetic Rats

**Figure 1:**
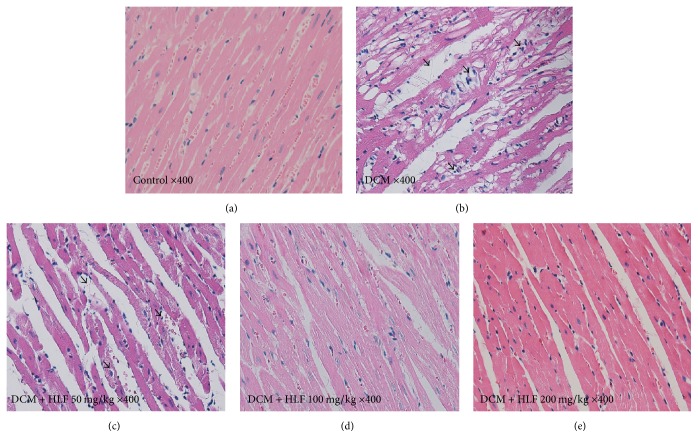
Effect of HLF on myocardial cell morphology in diabetic rats. (a) Control group; (b) DCM group; (c) DCM + HLF 50 mg/kg group; (d) DCM + HLF 100 mg/kg group; (e) DCM + HLF 200 mg/kg group; arrows indicate the disorder of myocardial cell and nuclear vacuolar.

As shown in Figure 1, control group showed arranged orderliness, clear morphology of myocardial fibers, and integrated cytomembrane. In contrast, DCM group showed disorder of myocardial cell, loosened junction between cells, dim myocardial fibers, and vacuolar degeneration. Interesting, HLF treatment reversed these changes obviously, especially in HLF high dose treatment group.

### 3.6. The Effect of HLF on the Expression of PKC-*α* mRNA in Myocardial Cell

**Figure 2:**
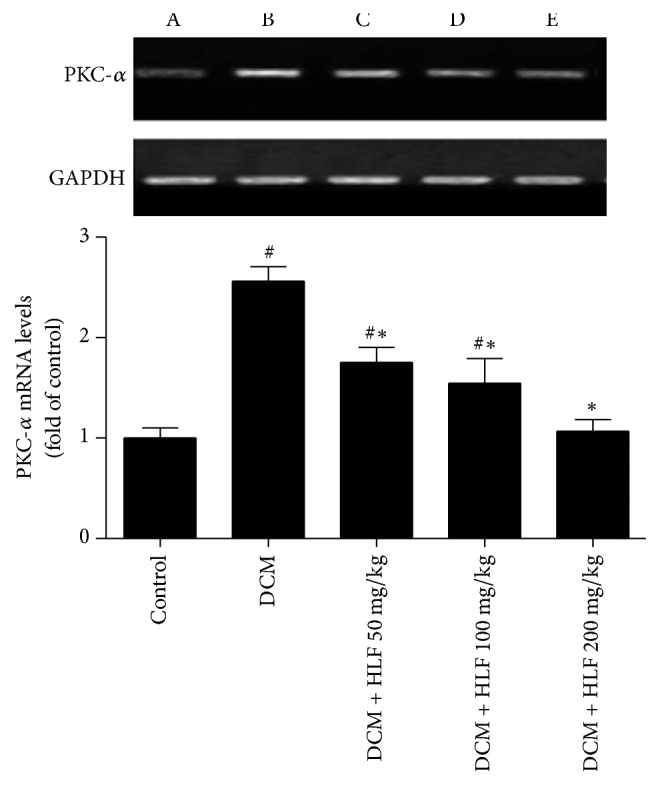
HLF reduced PKC-*α* mRNA expression on myocardial cell in diabetic rats. (A) Control group; (B) DCM group; (C) DCM + HLF 50 mg/kg group; (D) DCM + HLF 100 mg/kg group; (E) DCM + HLF 200 mg/kg group; data are mean ± SD. *n* = 3 per group; ^#^*P* < 0.05 versus control group; ^*∗*^*P* < 0.05 versus DCM group.

The result of PKC-*α* semiquantitative analysis in myocardial tissue was shown as Figure 2 and band of internal reference gene GAPDH and target gene were visible under the ultraviolet transmission reflection instrument. Varying degrees expressions of PKC-*α* mRNA in each experimental group could be seen. The expression of PKC-*α* mRNA was increased obviously in DCM group rats (*P* < 0.05 versus control group). However, HLF markedly inhibited the expression of PKC-*α* mRNA (*P* < 0.05 versus DCM group).

### 3.7. The Effect of HLF on the Expression of PKC-*α* in Myocardial Cell Cytoplasm and Membrane

**Figure 3:**
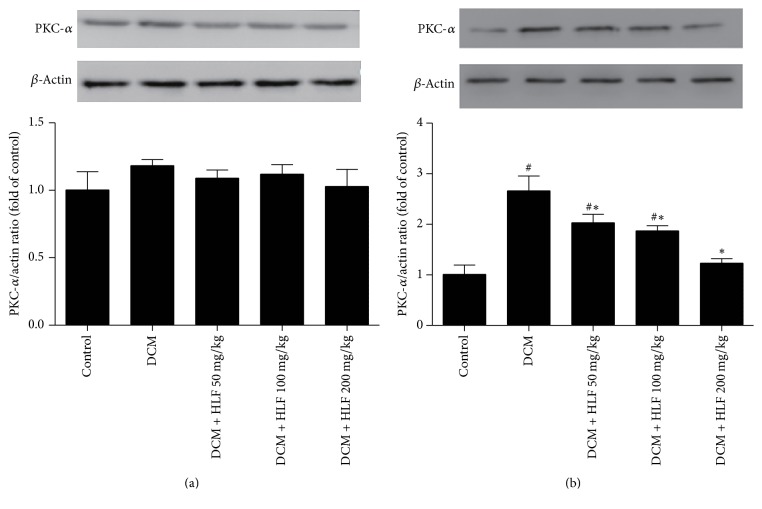
HLF regulated the PKC-*α* expression of cytoplasm and membrane on myocardial cell in diabetic rats. (a) Representative pictures of PKC-*α* expression of cytoplasm; (b) Representative pictures of PKC-*α* expression of membrane. Data are mean ± SD. ^#^*P* < 0.05 versus control group; ^*∗*^*P* < 0.05 versus DCM group; *n* = 3 per group.

As shown in Figure 3, PKC-*α* expression of myocardial cell cytoplasm has no significant difference in each of the groups, while the PKC-*α* expression of myocardial cell membrane markedly increased in DCM group rats compared with the control group (*P* < 0.05 versus control group). After treating with HLF, the protein expression of PKC-*α* was much lower than DCM group rats and this effect was shown in a dose-dependent manner (*P* < 0.05 versus DCM group).

### 3.8. The Effect of HLF on the Expression of NF-*κ*B p65 in Myocardial Cell Nucleus

**Figure 4:**
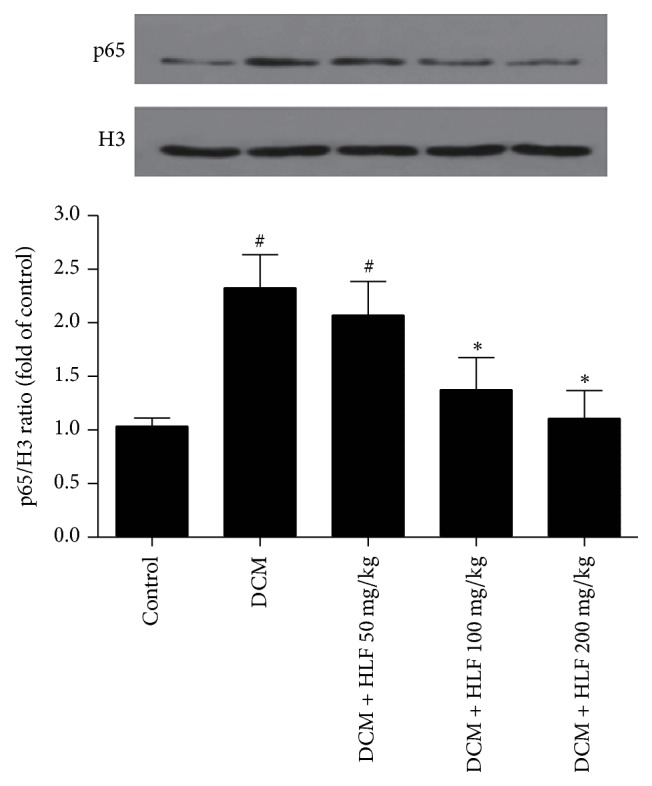
HLF reduced NF-*κ*B p65 expression of nucleus on myocardial cell in diabetic rats. Representative pictures reduced NF-*κ*B p65 expression of nucleus and quantitative analysis of NF-*κ*B p65. H3 served as the loading control. Data are mean ± SD. ^#^*P* < 0.05 versus control group; ^*∗*^*P* < 0.05 versus DCM group; *n* = 3 per group.

As shown in Figure 4, compared with the control group, NF-*κ*B p65 expression of myocardial cell nucleus in DCM group rats was remarkably increased (*P* < 0.05 versus control group); HLF 100 mg/kg and HLF 200 mg/kg remarkably restrained the increase of NF-*κ*B p65 induced by diabetes (*P* < 0.05). These results indicated that HLF could significantly inhibit the activation of NF-*κ*B p65 in myocardial cell nucleus.

## 4. Discussion

Diabetes is one of the most common metabolic disorders and can cause chronic complications including diabetic retinopathy, diabetic nephropathy, and neurologic disorder. Diabetic cardiomyopathy (DCM), another important complication, received more and more attention in recent years [14]. Myocardial damage induced by diabetes is independent of hypertension, coronary heart disease, and other cardiovascular diseases [15]. Diabetes can lead to metabolic disorder of myocardial tissue and influence the structure and function of the heart and aggravate heart failure by classical predisposing factor [16]. Diabetic patients accompanied by DCM are more likely to induce heart failure which is rapid progressive and has high mortality rate [17, 18]. However, the mechanisms of DCM are complex and have not been completely elucidated.

Diabetes can be divided into 2 types: type 1 and type 2 diabetes. The main characteristic of type 1 diabetes is the absolute destruction of pancreatic *β* cell and type 2 diabetes is insulin resistance accompanied by inadequate secretion. Type 2 diabetes accounts for 90 to 95% of diabetes cases [19]; thus type 2 diabetes animal model has an important role in the study of diabetes. High-fat diet combined with intraperitoneal injection of low dose of STZ is the most commonly used method to set up type 2 diabetic animal model [20]. In the present study, the living conditions of control group rats were favorable and the weight increases steadily. However, about 50% DCM group rats died due to the diabetic complication and showed the typical diabetic symptoms such as hyperglycemia, polydipsia, and polyuria. DCM group rats also showed the decreased body weight and increased HW/BW ratio, while, after the treatment of HLF, the above symptoms were improved. HLF treatment raised the body weight and declined the ratio of HW/BW and also significantly reduced the blood glucose level and showed a certain dose-dependent manner compared with the DCM group. These results indicated that HLF exerts a protective effect on DCM rats to some extent.

It is widely believed that DCM is associated with inflammation reaction, cell apoptosis, oxidative stress, the upregulated expression of extracellular matrix, and so on [21, 22]. Protein kinase C (PKC) activation is related to the regulation of protein phosphorylation. Furthermore, PKC is the committed step in the physiological effect [23]. PKC can induce the damage of myocardial tissue and blood vessels by some physiological effects such as promoting the synthesis of extracellular matrix, regulating the calcium ion metabolism of myocardial cell, activating Ang II, and inducing ROS and inflammatory factor. According to the researches [24–26], the activity of PKC in diabetic heart has increased even though the DCM myocardial tissue has not yet shown lesions of function and cardiac muscle. In the present study, we determined the mRNA and protein expression of PKC-*α* by PCR and western blot, respectively. These results showed that the expression of PKC-*α*mRNA in DCM group rats was significantly increased compared to that of the control group rats, while the expression in HLF treatment groups was downregulated obviously compared to the DCM group rats. Previous studies also have confirmed that the inactivation of PKC-*α* is present in the cytoplasm and will be transferred to membrane under the stimulation of high glucose; the membrane translocation of PKC-*α* is its symbol to be activated. Consistently, the protein expression of PKC-*α*, in myocardial cell cytoplasm and membrane, was measured in our study, and we found that the myocardial cell membrane expression of PKC-*α* in DCM group rats was markedly increased compared with the control group and significantly reduced after treatment with HLF. The above results demonstrated that HLF exerts these protective effects through inhibiting the expression of PKC-*α*.

Inflammation plays an important role in the occurrence and development of cardiovascular disease. The infiltration of inflammatory cells will promote the development of hypertension, atherosclerosis, myocardial ischemia, and chronic heart failure [27]. In the meantime, the inflammatory response is thought to be the main mechanism to promote the progression development of systemic disease in diabetic patients. In addition, inflammatory cytokines are involved in damaged heart tissue and significantly induced cardiac dysfunction after myocardial necrosis and myocardial infarction [28, 29]. In the diabetic patients, the elevation of TNF-*α* level in plasma is associated with myocardial diastolic dysfunction, and the activated NF-*κ*B can obviously increase the expression of TNF-*α* in left ventricular myocardium of diabetic rodents; on the other hand, TNF-*α* also upregulate NF-*κ*B expression. The interaction between TNF-*α* and NF-*κ*B will further aggravate the inflammatory lesions of myocardial tissue [30]. In the present study, the experimental results demonstrated that the expressions of NF-*κ*B p65 and TNF-*α* in myocardial tissue of diabetic rats hearts were remarkably upregulated and those expressions could be inhibited in HLF treatment. Our results exhibited that HLF has a certain anti-inflammatory effect and the mechanism may be related to inhibition of the PKC-*α* expression.

Accumulating lines of evidence have shown that the aggregation of intermediate oxidation product may be another pathogenic factor of DCM. A small amount of ROS produced by mitochondrial respiratory movement can be destroyed by antioxidants in the normal physiological condition. However, in the case of diabetes, the ROS will accumulate because antioxidants have lost too much and then the oxidative stress was enhanced, causing the damage of myocardial cell [22]. SOD is one of the most important biological antioxidant enzymes and is the primary material to eliminate free radical; the activity of SOD can reflect the body's antioxidant capacity [31]. MDA is the metabolite of lipid peroxidation damage, and it can be regarded as a symbol to evaluate the damage extent of cytomembrane which is induced by free radical [32]. In the present study, the activity of SOD in DCM group rats was markedly decreased and the content of MDA was markedly increased compared with the control group. Interestingly, after treating with HLF, the activity of SOD was increased and the content of MDA was significantly decreased. This demonstrated that HLF has an effect of antioxidant. The experiment results indicated that HLF could protect DCM rats by antioxidant intervention. Therefore, we considered that HLF can reduce the generation of ROS and alleviate the oxidative stress damage of DCM by inhibiting the expression of PKC-*α*.

Taken together, we found that HLF can protect against diabetes-induced cardiomyopathy and these effects may be through alleviating inflammatory and oxidative stress and suppressing activation of PKC-*α*.

## Figures and Tables

**Table 1:** Effect of HLF on metabolism in diabetic rats.

Group	Blood glucose levels	BW (g)	HW (g)	HW/BW (mg/g)
Control	5.0 ± 0.9	538 ± 33	1.34 ± 0.04	2.49 ± 0.08
DCM	20.0 ± 1.4^#^	280 ± 19^#^	1.00 ± 0.09^#^	3.58 ± 0.12^#^
DCM + HLF 50 mg/kg	14.3 ± 1.0^*∗*#^	319 ± 49^#^	1.04 ± 0.1^#^	3.28 ± 0.19^#^
DCM + HLF 100 mg/kg	10.4 ± 0.9^*∗*#^	359 ± 17^#^	1.12 ± 0.09	3.11 ± 0.13^*∗*#^
DCM + HLF 200 mg/kg	9.4 ± 1.0^*∗*#^	385 ± 20^*∗*#^	1.12 ± 0.08	2.93 ± 0.27^*∗*^

BW: body weight; HW: heart weight. Body weight and heart weight were determined on the day which the rat was killed. Data are mean ± SD; ^#^*P* < 0.05 versus control group; ^*∗*^*P* < 0.05 versus DCM group; *n*  =  8–10 per group.

**Table 2:** Effect of HLF on MDA content and SOD activity in diabetic cardiomyopathy.

Group	SOD U/mg protein	MDA nmol/mg protein
Control	91.7 ± 5.7	1.6 ± 0.1
DCM	80.5 ± 6.1	3.1 ± 0.3^#^
DCM + HLF 50 mg/kg	90.5 ± 5.4	2.6 ± 0.3^*∗*#^
DCM + HLF 100 mg/kg	103.4 ± 7.1^*∗*^	2.0 ± 0.2^*∗*^
DCM + HLF 200 mg/kg	104.5 ± 10.5^*∗*^	1.9 ± 0.1^*∗*^

Data are mean ± SD; ^#^*P* < 0.05 versus control group; ^*∗*^*P* < 0.05 versus DCM group; *n*  =  8–10 per group.

**Table 3:** Effect of HLF on TNF-*α* in diabetic cardiomyopathy.

Group	TNF-*α* pg/mg
Control	290 ± 26
DCM	698 ± 53^#^
DCM + HLF 50 mg/kg	591 ± 39^*∗*#^
DCM + HLF 100 mg/kg	483 ± 33^*∗*#^
DCM + HLF 200 mg/kg	419 ± 24^*∗*#^

Data are mean ± SD; ^#^*P* < 0.05 versus control group; ^*∗*^*P* < 0.05 versus DCM group; *n*  =  8–10 per group.
